# Therapeutic vaccination with IDLV-SIV-Gag results in durable viremia control in chronically SHIV-infected macaques

**DOI:** 10.1038/s41541-020-0186-5

**Published:** 2020-05-08

**Authors:** Maria Blasi, Elizabeth C. Wescott, Erich J. Baker, Benjamin Mildenberg, Celia LaBranche, Wes Rountree, Barton F. Haynes, Kevin O. Saunders, M. Anthony Moody, Donatella Negri, Sampa Santra, Andrea Cara, Mary E. Klotman

**Affiliations:** 1 Department of Medicine, Duke University Medical Center, Durham, NC USA; 2 Duke Human Vaccine Institute, Duke University Medical Center, Durham, NC USA; 3 Beth Israel Deaconess Medical Center, Boston, MA USA; 4 Department of Surgery, Duke University Medical Center, Durham, NC USA; 5 Department of Pediatrics, Duke University Medical Center, Durham, NC USA; 6 Department of Infectious Diseases, Istituto Superiore di Sanità, Rome, Italy; 7 National Center for Global Health, Istituto Superiore di Sanità, Rome, Italy; 8 Present Address: Department of Pathology, Microbiology, and Immunology, Vanderbilt University School of Medicine, Nashville, TN USA; 9 Present Address: Harvard Law School, Cambridge, MA USA

**Keywords:** HIV infections, Vaccines, HIV infections

## Abstract

Despite incredible scientific efforts, there is no cure for HIV infection. While antiretroviral treatment (ART) can help control the virus and prevent transmission, it cannot eradicate HIV from viral reservoirs established before the initiation of therapy. Further, HIV-infected individuals reliably exhibit viral rebound when ART is interrupted, suggesting that the host immune response fails to control viral replication in persistent reservoirs. Therapeutic vaccines are one current approach to improving antiviral host immune responses and enhance long term virus control. In the present study, we used an integrase defective lentiviral vector (IDLV) expressing SIV-Gag to boost anti-Gag specific immune responses in macaques chronically infected with the tier-2 SHIV-1157(QNE)Y173H. A single immunization with IDLV-SIV-Gag induced durable (>20 weeks) virus control in 55% of the vaccinated macaques, correlating with an increased magnitude of SIV-Gag specific CD8+ T-cell responses. IDLV-based therapeutic vaccines are therefore an effective approach to improve virus specific CD8+ T-cell responses and mediate virus control.

## Introduction

While antiretroviral therapy (ART) has had a significant impact on morbidity and mortality associated with HIV infection, HIV-positive individuals on chronic ART remain at increased risk of diseases associated with chronic ART and aging.^1^ The development of alternative therapeutic strategies that can provide durable suppression of viremia in the absence of ART and potentially reduce the size of the viral reservoir is a high priority.^2^ The observation that the induction of strong, functional HIV-specific CD8+ T-cell responses in some HIV-positive patients (elite controllers) results in durable control of HIV replication without ART,^3,4^ prompted the development of therapeutic vaccines that can induce similar T-cell responses with high frequency and greater breadth. However, approaches including whole inactivated virus or recombinant protein have demonstrated limited ability to increase HIV-specific responses, with poor immunogenicity and no clear impact on viral load.^5^ New approaches based on DNA and recombinant viruses have been developed and tested in clinical trials.^6–9^ Although most of these new vectors induced HIV-specific immune responses, they demonstrated limited efficacy in controlling viral replication. Preliminary studies using dendritic cell (DC)-based vaccines have shown some promising results,^10–12^ suggesting that a therapeutic vaccine that targets those antigen presenting cells might be beneficial. Most DC-based vaccine strategies tested so far used ex-vivo approaches, where DCs differentiated from patient derived monocytes were pulsed with synthetic peptides in the presence of activating cytokines and reinfused in the patient to activate antigen-specific cytotoxic T lymphocytes (CTLs). A limitation of these approaches is the short-term antigen presentation provided by peptide pulsing or exogenous antigen-loading strategies. A strategy to overcome these limitations is the use of an antigen delivery platform that can effectively transduce DCs in vivo and provide effective and durable antigen presentation.

Our group has developed both HIV- and SIV-based integrase defective lentiviral vectors (IDLV) to deliver a broad range of proteins for the induction of durable antigen specific immune responses in both mice and nonhuman primates (NHPs).^13–19^ SIV-based IDLV are more efficient at transducing both human and macaque DCs compared to an HIV-based IDLV.^20^ Here, we report the results of the preclinical evaluation of an SIV-based IDLV therapeutic vaccine in combination with an HIV-based IDLV expressing the broadly neutralizing antibody (bnAb) PGT121 in chronically simian human immunodeficiency virus (SHIV) infected NHPs. The objective of our study was to determine whether the combination of the two IDLV-based approaches could both boost SIV-Gag specific T-cell responses and produce the PGT121 bnAb in vivo, leading to suppression of SHIV viremia. Sustained virus control for >20 weeks was achieved in 55% of the vaccinated macaques. While injection of IDLV-PGT121 resulted in detectable very low-levels of the bnAb in plasma, a single immunization with IDLV-SIV-Gag boosted antigen specific CD4+ and CD8+ T-cell responses and this was sustained over 20 weeks post vaccination. Depletion of CD8+ cells resulted in rapid virus rebound, demonstrating an inverse correlation between the induction of CD8+ cell responses by IDLV-SIV-Gag vaccination and viremia. These data support the use of IDLVs in therapeutic approaches against HIV to induce antigen-specific immune responses that can durably contain virus replication.

## Results

### SHIV-1157(QNE)Y173H challenge outcome

**Fig. 1:**
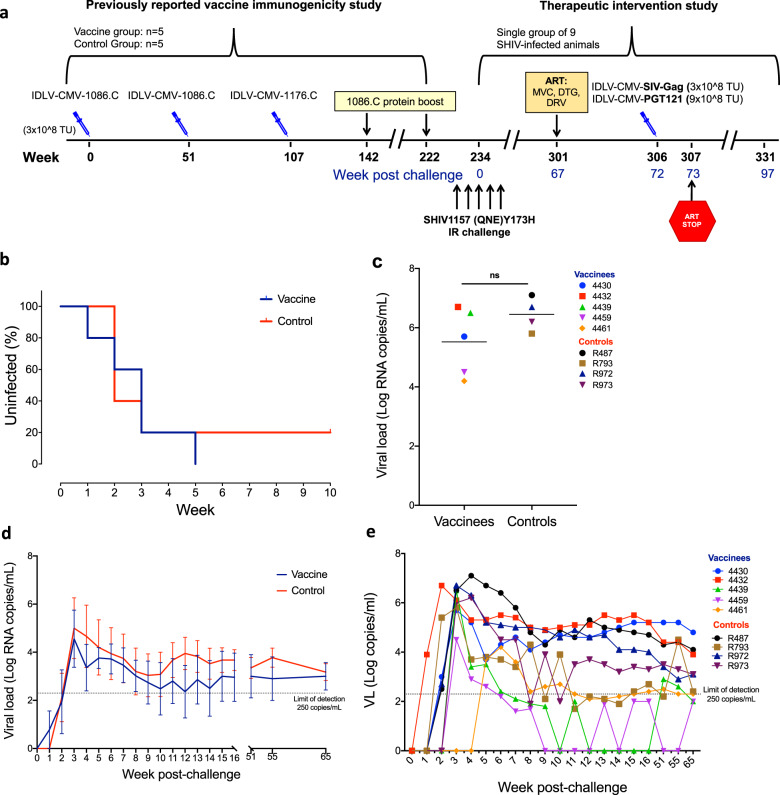
SHIV-challenge outcome and viral load trends in infected macaques. **a** Schematic of the preventive vaccine, SHIV-challenge and therapeutic intervention regimens. The nine macaques used in the therapeutic study reported here were previously employed in a preventive vaccine study testing the immunogenicity of an SIV-based IDLV expressing two clade C HIV-1 ENVs^16,19^. Twelve weeks after completion of the scheduled vaccinations (week 234) the 5 macaques in the vaccine group and the 5 macaques in the control group were challenged intrarectally with the Clade C tier 2 SHIV1157(QNE)Y173H. Weeks post challenge are indicated in blue. **b** Kaplan–Meier plot showing the percentage of uninfected macaques after 5 weekly IR challenges. **c** Peak viral load of the infected macaques from the vaccine and control groups. Lines are group means and error bars indicate s.e.m. (*p* = 0.5952, Exact-Wilcoxon test). **d** Viral load tested weekly after initial infection in both groups up to 65 weeks post infection (no significant difference was observed between the two groups, *p* = 0.7302, Exact Wilcoxon test). Lines indicate group means and error bars indicate s.e.m. **e** Individual animals viral load. The one animal in the control group that resisted infection is not shown and was not included in the therapeutic study.

Five Indian-origin rhesus macaques previously employed in the evaluation of the immunogenicity of a preventive IDLV-based vaccine expressing two clade C HIV-1 envelopes (1086.C and 1176.C),^16,19^ were challenged weekly intrarectally (IR) with low-dose heterologous Tier-2 SHIV-1157(QNE)Y173H^21^ (Fig. 1a). A group of five unvaccinated macaques were challenged as the control arm. Following five weekly IR challenges all but one animal in the control arm became infected (Fig. 1b). There was no difference in virus acquisition between the vaccinated or control group macaques (Fig. 1b). We observed a lower peak viremia (Fig. 1c) and lower mean viral loads over time (Fig. 1d) in the vaccine group compared to the control group, however, those differences were not statistically significant (*p* = 0.5952 and *p* = 0.7302, respectively, Exact Wilcoxon test). The majority of the infected animals (8/9) demonstrated durable viremia up to 65 weeks post challenge (Fig. 1e) and the mean viral load between the vaccine and control groups was the same at week 65 post challenge (3 vs. 3.1 log RNA copies/mL, respectively). These results indicate that the vaccine regimen previously administered to these macaques was not effective in preventing virus acquisition from repeated heterologous intrarectal challenges.

### Antibody and T-cell responses post challenge

**Fig. 2:**
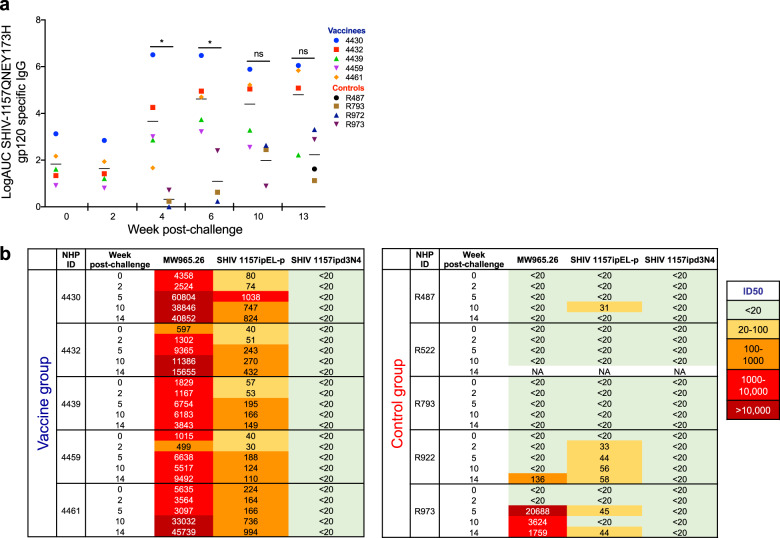
Binding and neutralizing antibody responses in SHIV-1157QNEY173H infected macaques. **a** ELISA binding of plasma antibodies to the SHIV encoded Env over the course of challenge for the vaccine and control group macaques. Binding titers measured as Log area under curve (Log AUC) starting at a 1:3000 plasma dilution. Horizontal bars are group means and error bars indicate s.e.m. Asterisks indicate *p* values < 0.05. **b** Antibody neutralization of Tier-1 viruses (MW965.26 and SHIV1157ipEL-p) and Tier-2 virus (SHIV-1157ipd3N4) measured in the TZM-bl neutralization assay as ID50. Values are the serum dilution at which relative luminescence units (RLUs) were reduced 50% compared to virus control wells (no test sample). The one animal in the control group that resisted infection (monkey ID: R522) was not included in the therapeutic study.

**Fig. 3:**
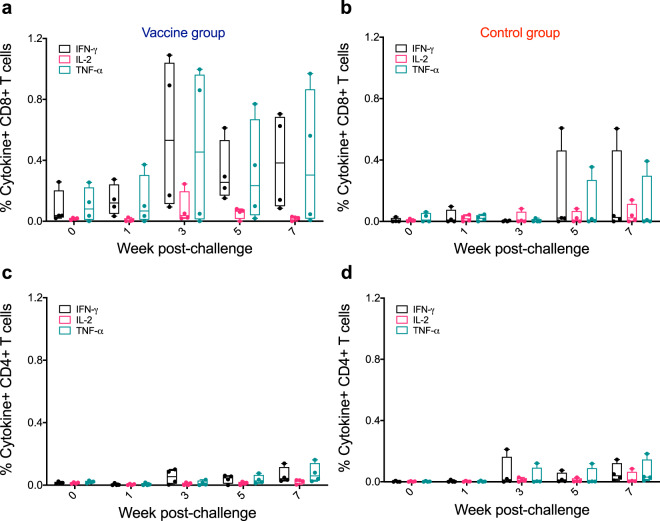
SIV-Gag specific CD4+ and CD8+ T-cell responses in SHIV-1157(QNE)Y173H infected macaques. The frequency of SIV-Gag-specific CD8+ (**a**, **b**) and CD4+ (**c**, **d**) T-cells expressing the cytokines IFN-γ, IL-2, and TNF-α was determined over time using cryopreserved PBMC stimulated overnight with SIV-Gag peptide pools.

We next measured the plasma antibody levels against the SHIV-1157(QNE)Y173H HIV-envelope (Env) encoded by the challenge SHIV (Fig. 2a). As expected, the group of macaques previously vaccinated with IDLV-Env had higher antibody responses to SHIV-1157(QNE)Y173H gp120 compared to the control group at the early time points (*p* = 0.0357 at both weeks 4 and 6). The magnitude of Env-specific antibody responses continued to increase over time in both groups, with no statistically significant difference between the two groups at later time points (*p* = 0.0714 at week 10 and *p* = 0.1143 at week 13). Tier 1 neutralizing antibody titers were also higher in the vaccine group compared to the control group (Fig. 2b), however, none of the macaques developed neutralizing antibodies against the parental tier 2 SHIV-1157ipd3N4 (Fig. 2b). Similarly, SIV-Gag specific CD8+ T-cell responses were higher in the vaccine group compared to the control group at early time points (Fig. 3a, b), while no difference in the percentages of cytokine positive CD4+ T-cells was observed (Fig. 3c, d). The higher magnitude of SIV-Gag specific CD8+ T-cell responses in the vaccine group at early time points post challenge is not surprising, given the presence of Gag in the IDLV-particles used for immunization. These data suggest that the vaccine-induced immune responses increased following SHIV acquisition, however those responses had no significant impact on virus replication over time.

### IDLV-SIV-Gag therapeutic vaccine increased the magnitude of SIV-Gag specific T-cell responses

**Fig. 4:**
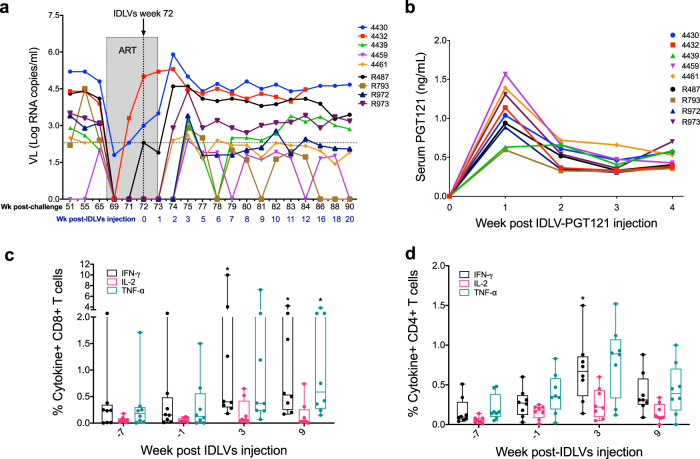
Viral load dynamics and SIV-Gag specific T-cell responses pre- and post-IDLV therapeutic interventions. **a** Plasma viral RNA levels were assessed before and after IDLVs injection. **b** Serum levels of PGT121 bnAb post-IDLV-PGT121 injection. Frequency of SIV-Gag-specific CD8+ **c** and CD4+ **d** T-cells expressing the cytokines IFN-γ, IL-2, and TNF-α were measured before and after IDLV-SIV-Gag vaccination. Note the difference in scale for **e** and **d** and the plots in Fig. 3. Asterisks indicate *p* values <0.05. Comparison were made between week −1 and week 3 or 9.

We next asked whether an IDLV-based therapeutic approach could have an impact on virus replication in these macaques chronically infected with SHIV-1157(QNE)Y173H for 67 weeks. We engineered an HIV-based IDLV to express the broadly neutralizing antibody (bnAb) PGT121 and an SIV-based IDLV to express the SIV-Gag protein. We used the SIV-based vector to deliver SIV-Gag because of the higher DC transduction efficiency of this vector compared to the HIV-based one, due to the presence of SIV-Vpx.^20^ Conversely, the HIV-based one was chosen to deliver the PGT121 bnAb to reduce DC transduction and the consequent induction of anti-PGT121 responses. Before injecting the two IDLV vectors, the macaques were treated with combination ART (maraviroc, dolutegravir, and darunavir) for 5 weeks. We did not include a reverse transcriptase inhibitor in the ART formulation as that would have also impacted IDLV reverse transcription. As shown in Fig. 4a and Supplementary Fig. 1, at 1 week after ART initiation, all the macaques had an undetectable viral load, however, at 5 weeks post ART initiation, there was detectable viremia in three of the nine macaques. ART was interrupted 1 week post IDLV-SIV-Gag and IDLV-PGT121 injection and viral loads, anti-SIV-Gag T-cell responses and PGT121 plasma levels were measured over time. Viremia was observed in all the macaques between 1 and 2 weeks post ART interruption, however, at 5 weeks post IDLV injection viral loads dropped below the limit of detection in five out of nine macaques (Fig. 4a). Among these five animals three had been previously vaccinated with IDLV-Env and two belonged to the challenge control group (not vaccinated with IDLV-Env).

To assess the serum levels of IDLV-produced PGT121 bnAb and to measure the IDLV-SIV-Gag induced T-cell responses, we performed ELISA and intracellular cytokine staining (ICS), respectively on samples collected before and after injection of the IDLVs. As shown in Fig. 4b, the PGT121 antibody was detected in the serum of all the IDLV-PGT121 injected macaques, however, the antibody levels were very low, ranging from 0.5 to 1.5 ng/mL. However, a robust and significant increase in the percentage of IFN-γ and TNF-α secreting Gag-specific CD8+ T-cells was observed at weeks 3 (*p* = 0.0379 for IFN-γ) and 9 (*p* = 0.0406 for IFN-γ and *p* = 0.0305 for TNF-α) post-IDLV-SIV-Gag injection compared to weeks 7 and 1 before IDLV-SIV-Gag immunization (Fig. 4c, Supplementary Fig. 2a–c). The percentage of cytokine+ CD4+ T-cells also increased following IDLV-SIV-Gag injection (*p* = 0.0207 for IFN-γ at week 3) (Fig. 4d, Supplementary Fig. 2d–f), although the magnitude of those responses was substantially lower compared to the CD8+ T-cell responses.

### Inverse correlation between viral load and magnitude of SIV-Gag specific CD8+ T-cell responses

**Fig. 5:**
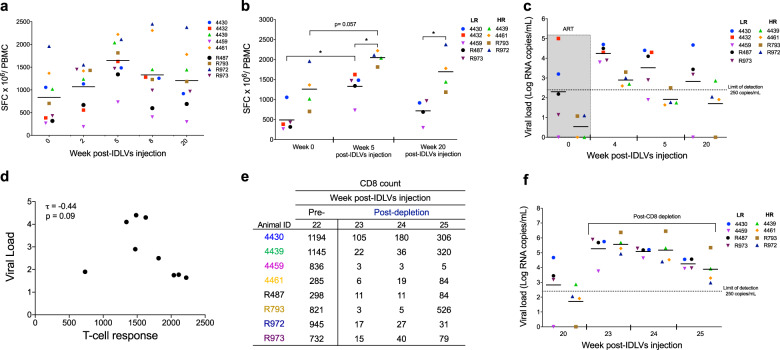
Inverse correlation between viral load and SIV-Gag specific CD8+ T-cell responses in IDLV injected macaques. **a** IDLV-induced T-cell responses were measured over time by IFN-Ɣ ELISpot assay. **b** Magnitude of T-cell responses in high responder (HR) vs. low responders (LR) macaques before and after IDLV-SIV-Gag immunization**. c** Plasma viral RNA levels post-IDLV vaccination in high responder (HR) vs. low responders (LR) macaques before and after IDLV-SIV-Gag immunization**. d** Kendall’s tau correlation between T-cell responses and viral load at week 5 post IDLV-SIV-Gag immunization. **e** CD8+ cell counts before and after administration of the CD8-depleting mAb cM-T807. **f** Plasma viral RNA levels post CD8+ lymphocytes depletion in high responder (HR) vs. low responders (LR) macaques. Asterisks indicate *p* values < 0.05.

We next performed IFN- γ ELISPOT assay to measure overall T-cell responses over time. The group of macaques previously vaccinated with IDLV-Env (animal IDs: 4430, 4432, 4439, 4459, and 4461) did not exhibit better T-cell responses following IDLV-SIV-Gag vaccination compared to the group of macaques not vaccinated with IDLV-Env (animals IDs: R487, R793, R972, and R973) (Fig. 5a). Anti-SIV-Gag specific T-cell responses peaked at 5 weeks post therapeutic vaccination which corresponded to the decline in viral load (Fig. 5a–c), suggesting an inverse correlation between the magnitude of anti-SIV-Gag specific T-cell responses and viral loads (Fig. 5d) (Kendall’s tau = −0.44). This phenomenon persisted for over 20 weeks (Kendall’s tau = −0.18) (Fig. 5a–c). Due to the small sample size *p* values did not reach statistical significance (*p* = 0.0953 at week 5 and *p* = 0.5330 at week 20). However, as shown in Fig. 5b, there was a significant difference in the magnitude of T-cell responses between the group of macaques that controlled viral load (high responders, HR) and the ones that did not (low responders, LR) following IDLV-SIV-Gag vaccination (*p* = 0.0159 at week 5 and *p* = 0.0286 at week 20). The magnitude of SIV-Gag specific T-cell responses at baseline was higher in the HR group, although not significantly different (*p* = 0.0635), and potentially resulted in higher T-cell responses post IDLV vaccination in this group compared to the LR group (Fig. 5b). This suggests that booster vaccinations may have the potential to increase the magnitude of T-cell responses in macaques with lower antigen specific T cells and induce virus control.

One of the macaques that demonstrated virus control (#4459) had the lowest T-cell response, suggesting that another mechanism(s) contributed to virus control in this animal.

To confirm the role of CD8+ lymphocytes in virus control, at 22 weeks post IDLV injection we depleted CD8+ lymphocytes using a single SC injection (20 mg/kg) of the CD8+ lymphocyte depleting monoclonal antibody (mAb) cM-T807 (Fig. 5e). As shown in Fig. 5f, following administration of mAb cM-T80, virus rebounded quickly in all the macaques, including animal 4459 that demonstrated virus control despite lower SIV-Gag specific T-cell responses. Viral load levels decreased again with the repopulation of CD8+ cells (Fig. 5e, f). These data demonstrate that anti-SIV-Gag specific CD8+ T-cell responses induced by the IDLV-SIV-Gag therapeutic vaccine controlled virus replication in chronically SHIV infected macaques.

## Discussion

Multiple lines of evidence have demonstrated that CD8+ T-cell responses to HIV and SIV infection inhibit virus replication.^22–24^ However, those virus-induced responses are unable to control the infection on their own in the vast majority of infected individuals and nonhuman primates. They do, however, suggest that enhancing these responses with a therapeutic vaccine could induce immunologic control of virus replication, thereby reducing the need for continuous ART.

Several therapeutic HIV vaccine candidates have been evaluated in clinical trials for immunogenicity and efficacy. However these trials have shown limited success in terms of HIV replication control in both acute^8,25^ and chronically^26^ infected individuals. Additional vaccine strategies need to be developed and tested to improve on those limited results.

In this study we demonstrate that a single immunization with an IDLV-SIV-Gag therapeutic vaccine enhanced the magnitude and functionality of SIV-Gag specific CD4+ and CD8+ T cells in chronically SHIV-1157(QNE)Y173H infected macaques which resulted in sustained suppression of plasma viremia in 55% of the vaccinated macaques. We observed an inverse correlation between the magnitude of T-cell responses and individual macaques’ viral load. The macaques that demonstrated better virus control post-IDLV-SIV-Gag vaccination had significantly higher T-cell responses compared to non controller macaques. The SIV-Gag specific T-cell responses increased significantly in all the vaccinated animals, but reached the magnitude required to mediate virus control only in the animals that had higher levels of SIV-Gag specific T cells at baseline (before IDLV-SIV-Gag immunization). These results suggest that booster vaccinations are required to further increase the magnitude of T-cell responses in macaques with lower antigen specific T-cells at baseline to induce virus control.

Importantly, the magnitude of the viral load increased in all the macaques after CD8+ lymphocyte depletion and decreased again upon CD8+ cell repopulation, supporting these cells as the main contributor to the observed virus suppression. These results are in line with previous studies demonstrating the impact of CD8+ T-cell depletion on viral load in SIV infected macaques.^27,28^

Although this study was conducted on macaques previously enrolled in a prophylactic vaccine study and did not include a control group of macaques (no therapeutic intervention), the clear boost in T-cell responses after IDLV-SIV-Gag vaccination associated with virus control and the loss of control following CD8+ lymphocytes depletion clearly demonstrate the contribution of the therapeutic vaccine to virus control.

Other therapeutic vaccines based on recombinant DNA (rDNA)^29^ and Ad26/MVA^30^ vectors have also demonstrated the ability to induce strong CD8+ T-cell responses in SHIV or SIV-infected macaques. However, in both studies the macaques received multiple doses of the vaccine, while the single dose of IDLV-SIV-Gag administered to the macaques in this study demonstrated similar T-cell responses. Additionally, those studies failed to demonstrate a clear association between the vaccine-induced responses and virus control. More promising results in terms of virus control were obtained in macaques that received Ad26/MVA therapeutic vaccines in combination with the toll-like receptor 7 (TLR7) stimulation.^30^ Thus, the addition of a TLR7 agonist to IDLV therapeutic vaccination may further enhance the virus control observed in our present study.

In addition to the IDLV-SIV-Gag vaccine, the macaques were injected with an HIV-based IDLV expressing the native heavy and light chains of the PGT121 bnAb. It is unlikely that the low amounts on PGT121 detected had any impact on the virus control observed in the injected macaques. Future studies will determine whether a higher vector dose and improved bnAb expression by IDLV will add to the control obtained in this study.

In summary, our study is a proof of concept that a single immunization with an IDLV-based therapeutic vaccine can enhance virus specific T-cell responses in chronically SHIV-infected macaques and induce prolonged virus suppression in the absence of ART. These results could be further improved with booster vaccinations and combinatorial approaches using optimized bnAb delivery or immune-modulatory molecules. Future studies will evaluate the durability of virus control following single and multiple IDLV therapeutic vaccine injections and the impact of IDLV therapeutic vaccination on the viral reservoir.

## Methods

### SHIV challenge of rhesus macaques

Macaques were challenged five times weekly by the intrarectal route with 1:10,000 dilution of our SHIV-1157(QNE)Y173H challenge stock (3.67 × 10^9^ copies/ml of viral RNA and 4.88 × 10^8^/ml TCID50 as measured on TZM-bl cells).^21^ The virus stock was grown from PBMCs and lymph nodes cells of an infected macaque at peak viremia following mitogen stimulation and the stock was titrated in rhesus macaques to select the appropriate challenge dose. Sequence diversity of the viral stock was determined by single-genome amplification.^31^

SHIV plasma viral RNA measurements were performed at the Immunology Virology Quality Assessment Center Laboratory Shared Resource, Duke Human Vaccine Institute, Durham, NC as described.^31^

### Construction of IDLV-SIV-Gag and IDLV-PGT121 plasmids

The codon-optimized SIV-Gag gene was obtained from the p01-426 plasmid (NIH AIDS Reagent Program) and was cloned into a SIV-based self-inactivating lentiviral transfer vector^15^ downstream of the internal CMV promoter (pGAE-CMV-SIV-Gag-Wpre). The heavy and light chain of the native PGT121 bnAb were cloned into an HIV-based self-inactivating lentiviral transfer vector downstream of the internal CMV promoter (pTY2-CMV-PGT121-Wpre). The 2A self-cleaving peptide technology was used to express both the heavy and light chain genes from a single open reading frame.

The transfer vector pGAE-CMV-GFP-Wpre expressing the green fluorescent protein (GFP), the IN-defective packaging plasmids pAd-SIV-D64V for the SIV-based vector and pcHelp/IN- for the HIV-based vector, and the Vesicular Stomatitis virus envelope G protein (VSV-G) pseudotyping vectors from New Jersey serotype (pVSV.GNJ; accession number: V01214.1), have been previously described.^19,32^

### Vector production and validation

The human epithelium kidney 293T Lenti-X cells (Clontech Laboratories, Mountain View, CA) were maintained in Dulbecco’s Modified Eagles medium (Thermo Fisher Scientific, Waltham, MA) supplemented with 10% fetal bovine serum (GE Healthcare Life Sciences, HyClone Laboratories, South Logan, UT) and 100 units per ml of penicillin–streptomycin–glutamine (PSG) (Thermo Fisher Scientific). For production of recombinant IDLV, 3.5 × 10^6^ Lenti-X cells were seeded on 100 mm diameter Petri dishes and transfected with 12 µg per plate of a plasmid mixture containing transfer vector, packaging plasmid and VSV.G plasmid in a 2:4:2 ratio, using the JetPrime transfection kit (Polyplus Transfection Illkirch, France) following the manufacture’s recommendations. At 48- and 72-h post transfection, culture supernatants were cleared from cellular debris by low-speed centrifugation and passed through a 0.45 μm pore size filter unit (Millipore, Billerica, MA). Filtered supernatants were concentrated by ultracentrifugation for 2 h at 23,000 RPM on a 20% sucrose cushion. Pelleted vector particles were resuspended in 1× phosphate-buffered saline (PBS) and stored at −80 °C until further use. Each IDLV-SIV-Gag and IDLV-PGT121 stock was titered using a reverse transcriptase (RT) activity assay (RetroSys RT ELISA kit, Innovagen, Lund, Sweden) and the corresponding transducing units (TU) calculated by comparing the RT activity of each IDLV-SIV-Gag or IDLV-PGT121 stock to the RT activity of IDLV-GFP stocks with known infectious titers.^16^

### Macaques and IDLV-injection protocol

The ten Indian origin rhesus macaques (*Macaca mulatta*), used in this study were previously employed in the evaluation of the immunogenicity of a preventive IDLV-based vaccine expressing two clade C HIV-1 envelopes (1086.C and 1176.C).^16,19^ Thirty-five weeks after the heterologous boost with IDLV-1176.C gp140 Env, the animals received two protein boosts, 80 weeks apart, each consisting of 100 μg of 1086.C gp140 protein in 15% of STR8S-C adjuvant, formulated as previously described.^33^ The animals were housed at BIOQUAL, Inc. in accordance with the recommendations of the Association for Assessment and Accreditation of Laboratory Animal Care International Standards and with the recommendations in the Guide for the Care and Use of Laboratory Macaques of the United States—National Institutes of Health. The Institutional Animal Use and Care Committee of BIOQUAL approved these experiments (study # 17-016P). The MHC-I genotype of the animals is shown in Supplementary Table 1. When immobilization was necessary, the macaques were sedated by intramuscular injection with 10 mg kg^−1^ of ketamine HCl. All efforts were made to minimize suffering. Details of animal welfare and steps taken to ameliorate suffering were in accordance with the recommendations of the Weatherall report, “The use of nonhuman primates in research”. Macaques were housed in climate controlled facility with an ambient temperature of 21–25 °C, a relative humidity of 40–60% and a 12 h light/dark cycle. Macaques were socially housed when possible or individually housed if no compatible pairing could be found. Macaques also received appropriate environmental enrichment. The macaques were housed in suspended stainless steel wire-bottomed cages and provided with a commercial primate diet and fresh fruit twice daily, with water freely available at all times. Macaques were immunized once intramuscularly with IDLV-SIV-Gag with 3 × 10^8^ TU per animal in 0.7-ml injection volume divided into two sites (left and right thighs). Each animal also received 9 × 10^8^ TU (2.7-mL injection volume) of IDLV-PGT121 injected intramuscularly into 4 sites (left and right thighs and arms). Peripheral blood was obtained prior to IDLVs injection, weekly up to 14 weeks post IDLVs injection, bi-weekly from week 14 to week 22 and then weekly until the end of the study. One animal (#4432) was necropsied at week 15 post IDLVs injection due to weight loss, poor appetite and diarrhea not related to vaccine administration.

### Antiretroviral therapy

All rhesus macaques were put on ART 67 weeks post SHIV-1157(QNE)Y173H infection. The drug regimen consisted of 375 mg of darunavir (DRV) and 100 mg of maraviroc (MRV) administered orally twice a day and 2.5 mg/kg of dolutegravir (DTG) administered once a day by the subcutaneous (SC) route.

### Direct ELISAs

High binding EIA/RIA 384 well plates (Corning) were coated overnight with 2 µg ml^−1^ of 1157(QNE)-Y173H gp120 protein in coating buffer (KPL, Gaithersburg, MD). After one wash with washing buffer (1× PBS + 0.1% Tween 20) plates were treated for 1 h at room temperature with 40 μl per well of blocking buffer (PBS containing 4% [wt/vol] whey protein—15% normal goat serum—0.5% Tween 20). Serial 3-fold dilutions of plasma (from 1:3000 to 1:7,29,000) or monoclonal antibodies (from 100 μg mL^−1^ to 0.5 ng mL^−1^) in blocking buffer were added to the plates (10 μl per well) in duplicates and incubated for 1.5 h at room temperature. The Rhesus B12 IgG (b12R1) was used to develop standard curves (range 100–0.005 ng mL^−1^, with each dilution assayed in duplicate). Abs were detected by adding 10 μl per well of horseradish peroxidase-conjugated, polyclonal goat anti-monkey IgG (Rockland,Gilbertsville, PA, Cat# 617-103-012) diluted in blocking buffer (1:6000) and by adding 20 μl per well the SureBlue Reserve TMB microwell peroxidase substrate and stop solution (KPL, Gaithersburg, MD). Binding titers were analyzed as area-under-curve of the log transformed concentrations (Softmax Pro 7, Molecular Devices LLC, CA).

For PGT121 detection in monkeys’ sera, direct ELISAs were performed as described above with the following modifications: plates were coated with 100 µg ml^−1^ of the STA09 protein (scaffold protein displaying the V1V2-region and PGT121 epitope).^34^ The PGT121 mAb was used to develop standard curves at the dilutions indicated above.

### HIV neutralization assays

Neutralization of Env-pseudotyped viruses was measured in 96-well culture plates using Tat-regulated firefly luciferase (Luc) reporter gene expression to quantify reductions in virus infection in TZM-bl cells.^35^ Heat-inactivated (56 °C, 1 h) serum samples were assayed at threefold dilutions starting at 1:20 against a panel of viruses consisting of replication competent SHIVs (1157ipEL-p, tier 1, and 1157ipd3N4, tier 2) and HIV pseudotyped with the MW965.26 Env (tier 1). Neutralization titers (50% inhibitory dose (ID50)) are the serum dilutions at which relative luminescence units (RLU) were reduced by 50% compared to RLU in virus control wells after subtraction of background RLU in cell control wells. A response was considered positive for HIV neutralizing antibody activity if the ID50 was ≥3 times the ID50 of the day of challenge sera (week 0) against the same virus.

### IFN-γ ELISpot assay

Multiscreen ninety-six well plates were coated overnight with 100 μl per well of 5 μg/ml anti-human interferon-γ (IFN-γ) (Clone B27; Becton, Dickinson and Company, Franklin Lakes, NJ, Cat# 554699) in endotoxin-free Dulbecco’s-PBS (D-PBS). Plates were washed three times with D-PBS containing 0.1% Tween-20, blocked for 2 h with Roswell Park Memorial Institute 1640 medium (RPMI) containing 10% fetal bovine serum and incubated with peptide pools and 2 × 105 PBMCs in triplicate in 100 μl reaction volumes. Each peptide pool (1 μg/ml) was comprised of 15 amino acid peptides overlapping by 11 amino acids. The pools covered the entire SIV-Gag protein. Following 18-h incubation at 37 °C, plates were washed nine times with D-PBS containing 0.1% Tween-20 and once with distilled water. Plates were then incubated with 2 μg/ml biotinylated rabbit anti-human IFN-γ (U-CyTech biosciences, Utrecht, The Netherlands, Cat# CT243) for 2 h at room temperature, washed six times with D-PBS containing 0.1% Tween-20, and incubated for 1.5 h with a 1:500 dilution of streptavidin-AP (Southern Biotechnology, Birmingham, AL, Cat# 7100-04). After five washes with D-PBS containing 0.1% Tween-20 and three washes with D-PBS alone, the plates were developed with bromochloroindolyl phosphate–nitro blue tetra-zolium (BCIP-NBT) chromogen (Thermo Fisher Scientific), stopped by washing with tap water, air dried, and read with an ELISpot reader (Cellular Technology Limited (CTL), Cleveland, OH) using ImmunoSpotAnalyzer software (Cellular Technology Limited (CTL)). Samples were considered positive if number of SFC was above twice that of the background (unstimulated) and >50 SFC per million PBMC.

### Intracellular cytokine staining

PBMC were isolated from EDTA-anticoagulated blood (GE Healthcare Bio-Sciences, Pittsburgh, PA) and cryopreserved. Cells were later thawed and rested 4 h at 37 °C in a 5% CO_2_ environment. PBMC were then incubated for 6 h in the presence of either RPMI containing 10% fetal bovine serum (unstimulated), PMA/ionomycin as positive control, or pool of SIV Gag peptides. All cultures contained a protein transport inhibitor, monensin (Golgi Plug; Becton, Dickinson and Company), and 1 μg/ml of anti-CD49d (Becton, Dickinson and Company, Cat# 340976). Cultured cells were then stained with a cell viability marker (Fixable Live Dead Yellow, Thermo Fisher Scientific, Cat# L34959) and pre-titered quantities of anti-CD4 APC-H7 (L200; Becton, Dickinson and Company, Cat# 560837), anti-CD8 BV570 (RPA-T8, BioLegend, San Diego, CA, Cat# 301037), anti-CD28 PE Cy7 (28.2, eBioscience, Cat# 560684), and anti-CD197 PerCP-Cy5.5 (150503, Becton, Dickinson and Company, Cat# 561144). Following fixation and permeabilization with Cytofix/Cytoperm solution (Becton, Dickinson and Company), cells were stained with anti-CD3 V450 (SP34.2, Becton, Dickinson and Company, Cat# 560352), anti-CD69-ECD (TP1.55.3, Beckman Coulter, Brea, CA, Cat# 6607110), anti-IFN-γ APC (B27, Becton, Dickinson and Company, Cat# 554702), anti-TNF-α-FITC (MAb11, Becton, Dickinson and Company, Cat# 554512), and anti-IL-2 PE (MQ1-17H12, Becton, Dickinson and Company, Cat# 554566). Samples (500,000 events per sample) were analyzed on a LSR II instrument (Becton, Dickinson and Company, Franklin Lakes, NJ) using FlowJo software (version 9.3.1). Samples in which the percentage of cytokine-staining cells were at least twice that of the background or in which there was a distinct population of cytokine brightly positive cells were considered positive.

### Statistical analysis

Comparisons between groups or time points were made using the Exact Wilcoxon test because of the small sample size. No adjustment to the alpha level was made for multiple comparisons. For Fig. 1d, AUC for each animal were calculated for comparison purposes. All computations were made using SAS v9.4 (SAS Institute, Inc.).

### Reporting summary

Further information on research design is available in the Nature Research Reporting Summary linked to this article.

## Supplementary information

Supplementary Information

Reporting Summary

## Data Availability

The data that support the findings of this study are available from the corresponding authors upon request.
