# Supplementary material for: Therapeutic vaccination with IDLV-SIV-Gag results in durable viremia control in chronically SHIV-infected macaques
Source: NPJ Vaccines. 2020 May 8;5:36. doi: 10.1038/s41541-020-0186-5 (PMC7210278; doi:10.1038/s41541-020-0186-5)
Supplement: Supplementary file 1 — Supplementary Information [file 41541_2020_186_MOESM1_ESM.pdf]

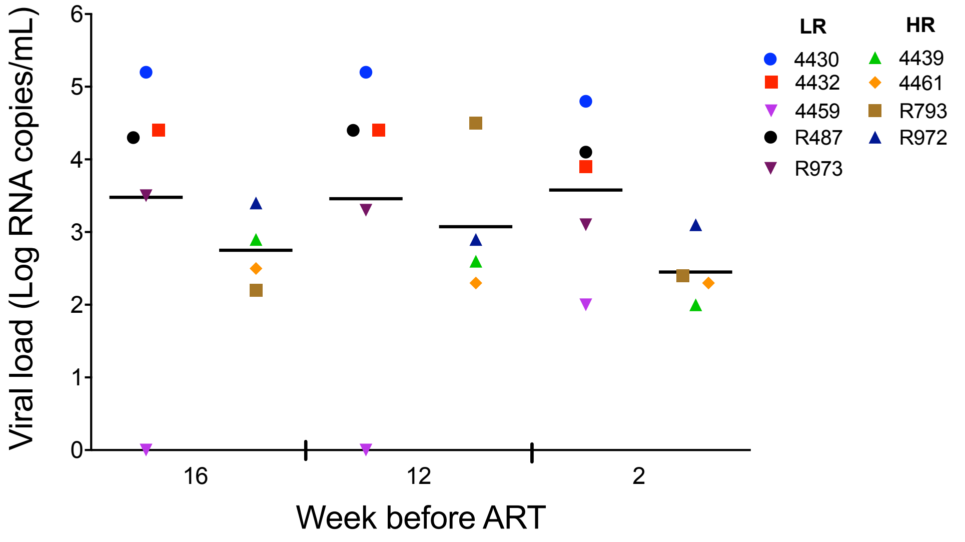

Supplementary Figure 1. Viral load levels in SHIV infected macaques before ART treatment. Plasma viral RNA levels in SHIV-infected animals was assessed at 16, 12 and 2 weeks before ART treatment. Animals are grouped based on their T cell responses (low responder vs high responder animals).

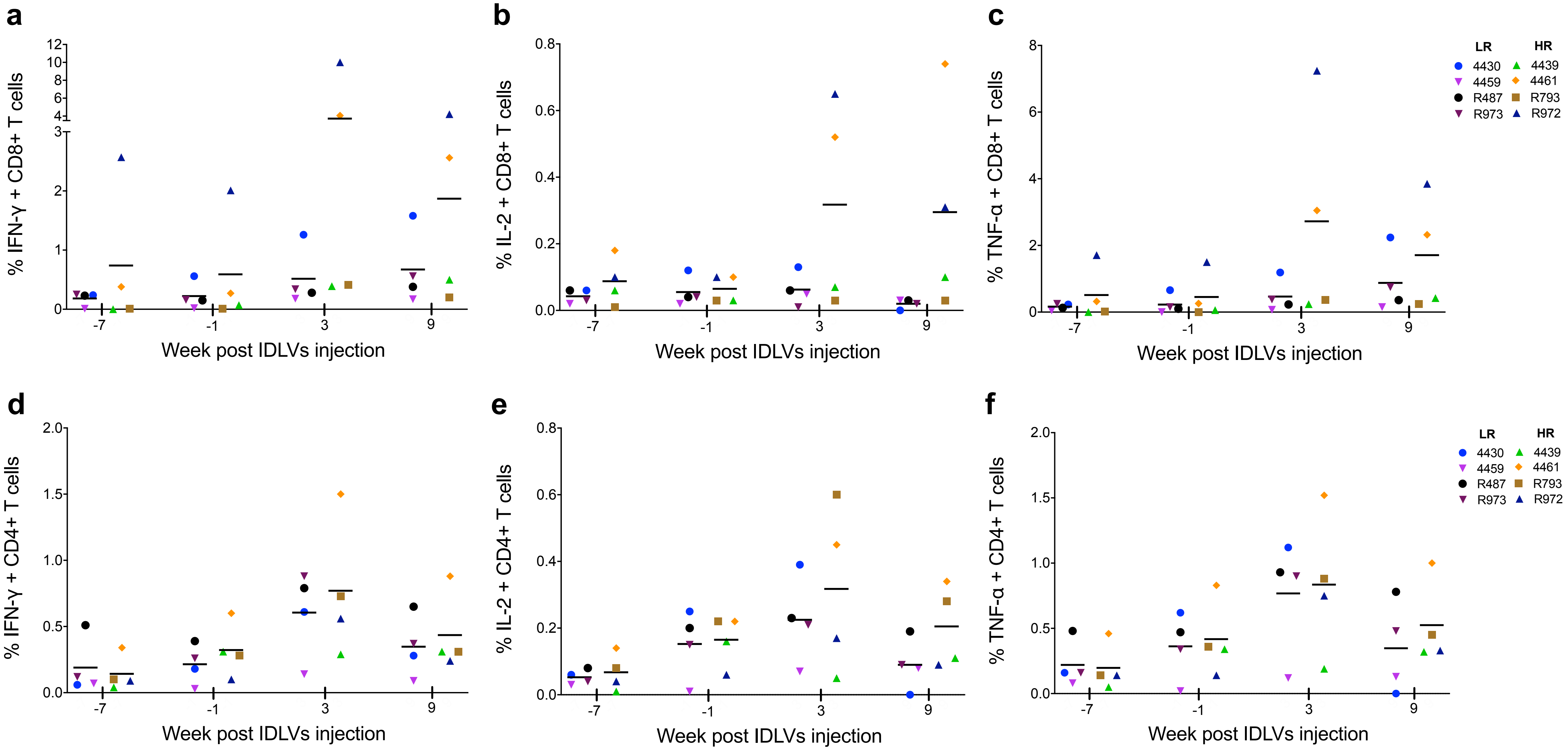

Supplementary Figure 2. SIV-Gag specific T cell responses pre- and post- IDLV therapeutic interventions. (a-c) Frequency of SIV-Gag-specific CD8+ and **(d-f)** CD4+ T-cells expressing the cytokines IFN-γ, IL-2, and TNF-α were measured before and after IDLV-SIV-Gag vaccination in high responder (HR) versus low responders (LR) macaques**.** Note the difference in scale for each graph.

Animal ID	MHC type
4428	N/A
4430	B01
4432	A08 B01
4439	N/A
4459	A02 A08 B01
4461	A02 A08
R487	N/A
R793	N/A
R972	N/A
R973	N/A

Supplementary Table 1. Macaques' MHC-I genotype. N/A = not available
